# Cardiac biomarkers of acute coronary syndrome: from history to high-sensitivity cardiac troponin

**DOI:** 10.1007/s11739-017-1612-1

**Published:** 2017-02-11

**Authors:** Pankaj Garg, Paul Morris, Asma Lina Fazlanie, Sethumadhavan Vijayan, Balazs Dancso, Amardeep Ghosh Dastidar, Sven Plein, Christian Mueller, Philip Haaf

**Affiliations:** 1 Leeds Institute of Cardiovascular and Metabolic Medicine (LICAMM), University of Leeds, Leeds, UK; 2 Cardiology and Cardiothoracic Surgery, Sheffield Teaching Hospitals NHS Foundation Trust, Herries Road, Sheffield, S5 7AU UK; 3 Department of Cardiovascular Science, University of Sheffield, Medical School, Beech Hill Road, Sheffield, S10 2RX UK; 4 Bristol Heart Institute, Bristol Royal Infirmary, Upper Maudlin Road, Bristol, BS28HW UK; 5 Department of Cardiology and Cardiovascular Research Institute Basel (CRIB), University Hospital Basel, Basel, Switzerland

**Keywords:** Cardiac biomarkers, High-sensitivity cardiac troponin, Acute coronary syndrome

## Abstract

The role of cardiac troponins as diagnostic biomarkers of myocardial injury in the context of acute coronary syndrome (ACS) is well established. Since the initial 1st-generation assays, 5th-generation high-sensitivity cardiac troponin (hs-cTn) assays have been developed, and are now widely used. However, its clinical adoption preceded guidelines and even best practice evidence. This review summarizes the history of cardiac biomarkers with particular emphasis on hs-cTn. We aim to provide insights into using hs-cTn as a quantitative marker of cardiomyocyte injury to help in the differential diagnosis of coronary versus non-coronary cardiac diseases. We also review the recent evidence and guidelines of using hs-cTn in suspected ACS.

## Introduction

A rapid and accurate diagnosis is critical in patients with presumed acute coronary syndrome for the initiation of effective evidence-based medical management and revascularization. The third universal definition of myocardial infarction defines an acute myocardial infarction (AMI) as evidence of myocardial necrosis in a patient with the clinical features of acute myocardial ischemia, and defines the 99th percentile of cardiac troponins as the decision value for AMI [1]. Clinical assessment, 12-lead ECG and cardiac troponin (cTn) I or T form the diagnostic cornerstones of patients with acute onset chest pain. Contemporary sensitive and high-sensitivity cardiac troponin (hs-cTn) assays have increased diagnostic accuracy in patients with acute chest pain in comparison with conventional cardiac biomarkers [2]. Rapid rule-in and rule-out diagnostic strategies for patients with chest pain in the emergency department (ED) are now available, and help clinicians to risk stratify patients and enable discharge of those deemed to be at very low risk. In principle, this improves assessment and makes ED care more cost effective. However, there is potential for confusion, misuse and unnecessary follow-up examinations when they are used imprudently [3]. Novel hs-cTn assays are able to quantify troponin in the majority of healthy individuals [4]. Although hs-cTn assays are very sensitive, they are less specific for AMI when using the 99th percentile as a single cutoff level [5]. Even when a troponin rise is consistent with a diagnosis of AMI, other cardiac diseases such as myocarditis, Tako-tsubo cardiomyopathy or shock can produce significant changes of troponin as well. Interpretation of the results is heavily dependent on the clinical context in which it is requested.

The aim of the current article is to review the history and evolution of cardiac biomarkers of acute coronary syndrome, define what troponin is, and aid in the use of the hs-cTn in clinical practice according to current guidelines.

## History of cardiac biomarkers

**Fig. 1:**
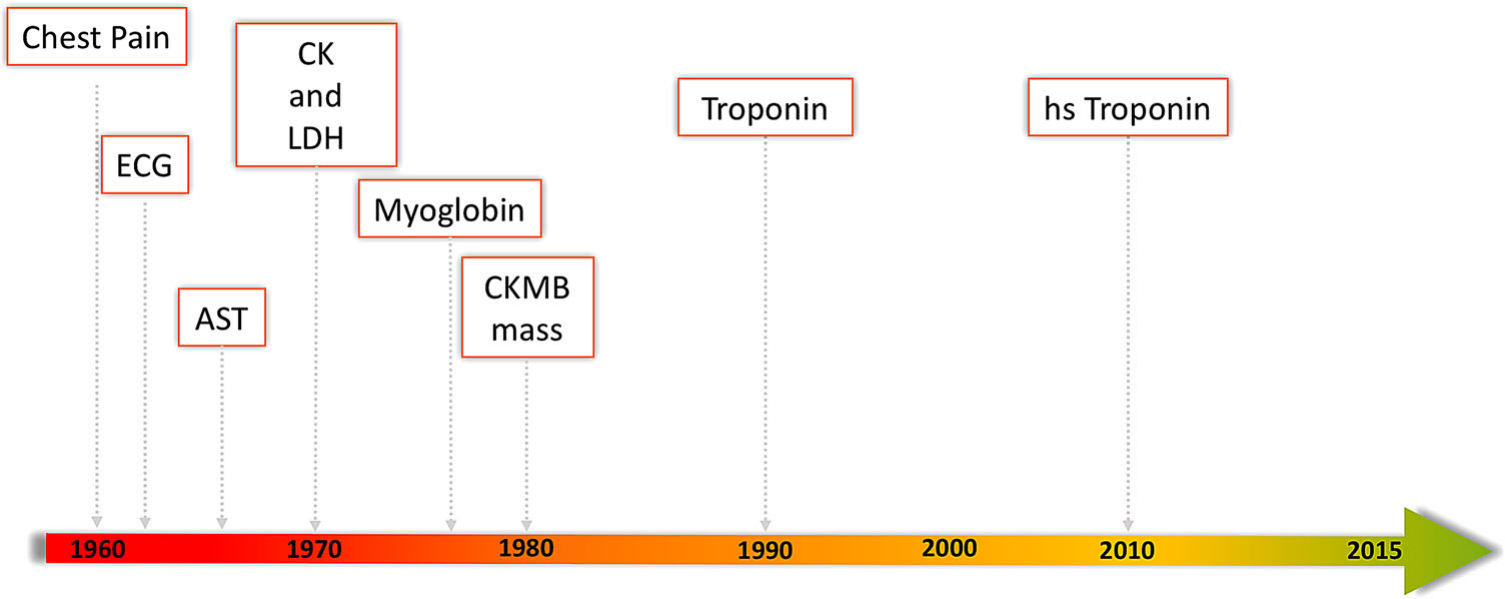
Timeline of the development of cardiac biomarkers for the diagnosis of acute myocardial infarction

Aspartate Transaminase (AST) became the first biomarker used in the diagnosis of AMI [6] (Fig. 1). AST was widely used in the 1960s and was incorporated into the World Health Organization (WHO) definition of AMI [7]. However, AST is not specific for cardiac muscle, and its detection is, therefore, not specific for cardiac damage. By 1970s, two further cardiac biomarkers were in use: lactate dehydrogenase (LDH) and creatine kinase (CK). Although neither is absolutely specific for cardiac muscle, CK is more specific than LDH in the context of AMI, especially in patients having other co-morbidities such as muscle or hepatic disease [8]. Myoglobin is a small globular oxygen-carrying protein found in heart and striated skeletal muscle [9]. The first method to detect myoglobin in the serum was developed in 1978. Myoglobin rises after acute myocardial injury, and it became a useful cardiac biomarker in the differential diagnosis of suspected AMI [10]. In the era of hs-cTn, testing of myoglobin as an early marker of myocardial necrosis is no longer recommended, neither as a single marker nor in a multi-marker strategy [11, 12]. Eventually, advancements in electrophoresis allowed the detection of cardiac-specific iso-enzymes of CK and LDH, i.e., CK-MB and LDH 1 + 2 [13]. Cardiac muscle has higher CK-MB levels (25–30%) compared to skeletal muscle (1%), which is mostly composed of CK-MM. These assays played an important role in the diagnosis of AMI for two decades, and were included as one of the diagnostic criteria to rule out AMI by the WHO in 1979 [14]. However, the lack of specificity and the high rate of false-positive results limited their usefulness. A more cardiac-specific biomarker was needed.

In 1965, a new protein constituent of the cardiac myofibrillar apparatus was discovered, which subsequently came to be known as troponin [15]. In the late 1990s, a sensitive and reliable radioimmunoassay was developed to detect serum troponin [16]. Numerous studies demonstrate that troponins appear in the serum 4–10 h after the onset of AMI [17]. Troponin levels peak at 12–48 h, but remain elevated for 4–10 days. The sensitivity for detecting troponin T and I approaches 100% when sampled 6–12 h after acute chest pain onset [18]. Therefore, in the context of acute chest pain, to reliably rule out AMI, patients need to have a repeat troponin sample 6–12 h after the initial assessment. Consequently, patients were increasingly admitted to observational chest pain units.

## What is troponin?

Troponin is a component of the contractile apparatus within skeletal and cardiac myocytes. Along with calcium ions, troponin proteins regulate and facilitate the interaction between actin and myosin filaments as part of the sliding filament mechanism of muscle contraction. Cardiac troponin (cTn) is a complex comprising three subunits:

- troponin T attaches the troponin complex to the actin filament;
- troponin C acts as the calcium binding site;
- troponin I inhibits interaction with myosin heads in the absence of sufficient calcium ions.

Troponin C is synthesised in skeletal and cardiac muscle. Troponin T and I isoforms are highly specific and sensitive to cardiac myocytes and, therefore, are known as cardiac troponins (cTn). The detection of cTn-T or cTn-I in the blood stream is, therefore, a highly specific marker for cardiac damage [19]. 92–95% of troponin is attached to the actin thin filaments in the cardiac sarcomere, and the remaining 5–8% is free in the myocyte cytoplasm [20]. Free, unbound cTn constitutes the ‘early releasable troponin pool’ (ERTP) [4]. The concept of the ERTP helps when considering the various mechanisms of troponin release into the blood stream. ERTP is thought to be released immediately following myocyte injury and, assuming normal renal function, this would be cleared promptly. This is contrary to the structurally bound cTn, which degrades over a period of several days causing a more stable and gradual troponin release.

The plasma half-life of cTn is around 2 h. Although the precise mechanism by which troponin is eliminated from the body remains unclear, it is hypothesized that troponin is cleared, at least in part, by the renal reticulo-endothelial system [21].

## High-sensitivity cardiac troponin

**Fig. 2:**
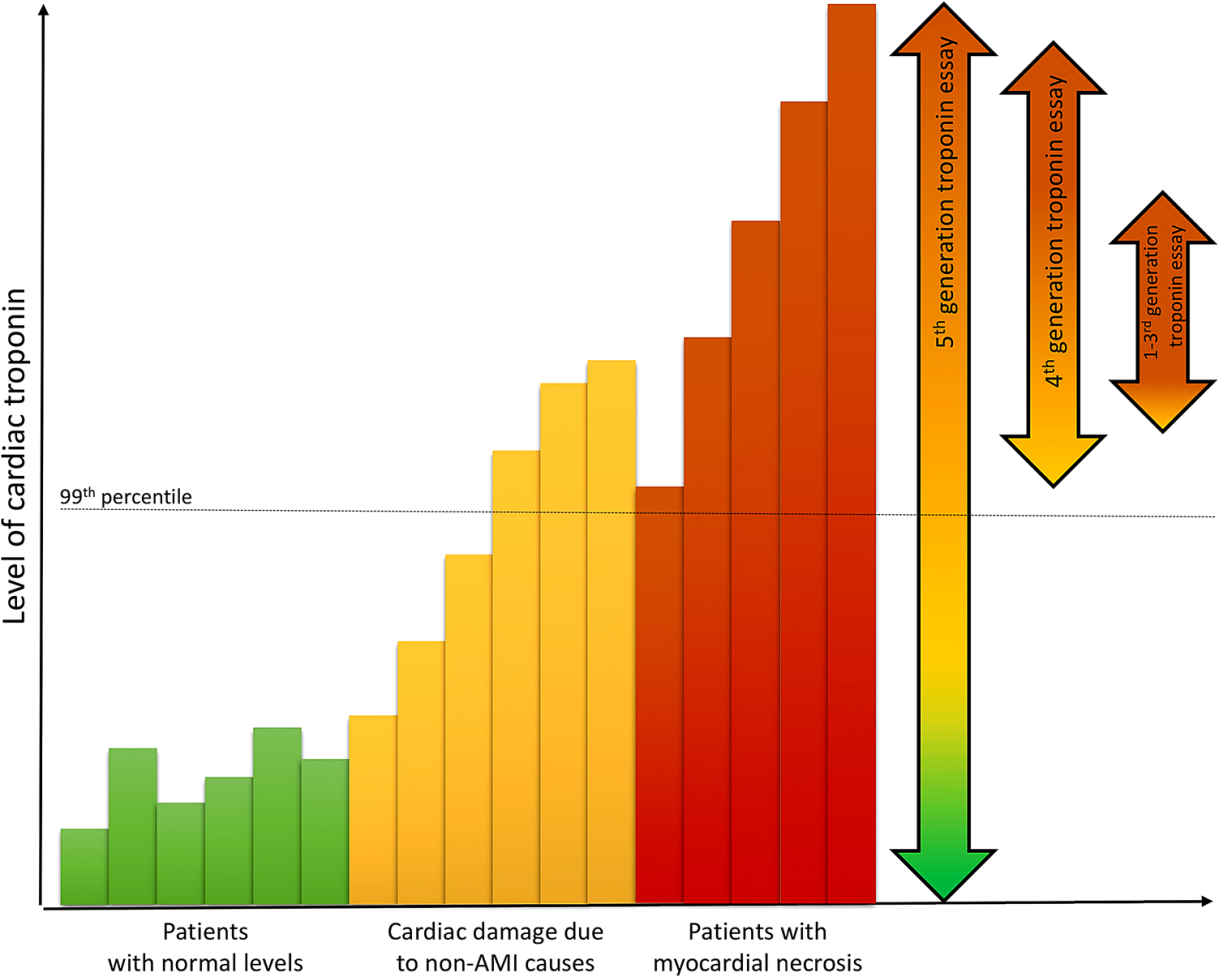
Detection range of different troponin assays. The *green bars* represent the normal turnover range of troponin in healthy individuals. With the onset of myocardial infarction, a slight rise in cardiac troponin can be seen that represents either ischemia-induced release of cytosolic troponin or micro-necrosis (*orange-bars*). Between 2 and 6 h, a steep increase in levels of cardiac troponin can be seen that represents extensive myocardial necrosis (*red-bars*). Only this major increase of cardiac troponin can be detected by first to fourth generation troponin assays. hs-cTn (5th generation troponin assay) can also detect lower levels of troponin including ischemia/micro-necrosis and even the normal turnover

Most hospitals now have replaced conventional cTn tests with the new 5th generation hs-cTn T and I assays which can detect troponin at concentrations 10- to 100-fold lower than conventional assays (Fig. 2). Various terms for “more sensitive” cTn assays have been proposed for marketing purposes. Troponin assays are recommended to be differentiated in conventional, sensitive and high-sensitivity cTn assays. Basically, hs-cTn assays detect troponin with higher sensitivity and precision at an earlier point of time [22], and allow detection and quantification in 50% to ideally 95% of healthy individuals (Table 1) [23]. Troponins are quantitative markers of cardiomyocyte injury, and the likelihood of AMI increases with increase in the level of cTn [24]. The negative predictive value (NPV) of hs-cTn assays is >95% for AMI exclusion when patients are tested on arrival at the ED [25]. If this is repeated at 3 h, this rises to nearly 100% [26]. Shah et al. demonstrated that using lower cutoffs for hs-cTn I (5 ng/L) identifies low-risk patients for the composite outcome of index myocardial infarction, and myocardial infarction or cardiac death at 30 days with an NPV of 99.6% (95% CI 99.3–99.8%) [27]. A recent systematic review and meta-analysis demonstrated that ‘lower cutoffs’ (3–5 ng/L versus 14 ng/L) for a single baseline hs-cTn T measurement improve sensitivity for AMI markedly, and can be used as a rule-out test in patients presenting more than 3 h after symptom onset [28]. Therefore, hs-cTn facilitates earlier exclusion of AMI, contributing to reduced ED length of stay, and earlier treatment for AMI resulting in improved outcomes [29]. However, the high sensitivity of these assays may result in increased numbers of patients with elevated hs-cTn levels being admitted for further assessment.

**Table 1:** Diagnostic characteristics of validated high-sensitivity cardiac troponin assays Adapted from Apple et al. [23]

Assay	LoD (ng/L)	99th % (ng/L)	% CV at 99th %	10% CV (ng/L)
hs-cTnT (Elecsys)	5	14	8	13
hs-cTnI (Architect)	1.2	16	5.6	3
hs-cTnI (Dimension Vista)	0.5	9	5	3

High-sensitivity cardiac troponin assays (5th generation)

*LOD* limit of detection, *CV* coefficient of variation

## Causes of hs-cTn elevation and risk of misinterpretation

There is a misconception that troponin elevation is secondary only to myocyte injury and necrosis. There are six mechanisms that have been proposed to explain the release of troponin into the bloodstream: normal cell turnover, myocyte necrosis, apoptosis or programmed cell death, proteolytic fragmentation, increased cell membrane permeability and membranous blebs.

Whether or not cTn is detectable, or even elevated, is therefore dependent on the balance of a host of interdependent factors, including the sensitivity of the test. Furthermore, there are potentially other, as yet not described mechanisms involved in the release of cTn. For example, it is still unknown why cTn is elevated in certain extra-cardiac disease processes such as sepsis. Whether ischemia causes elevated cTn in the absence of myocyte necrosis remains controversial [30]. Some animal and human studies demonstrate an association between reversible ischemia (no evidence of MI) and cTn elevation [31], whereas others fail to do so [32]. Increased myocardial strain is also considered to be associated with cTn elevation [33].

**Fig. 3:**
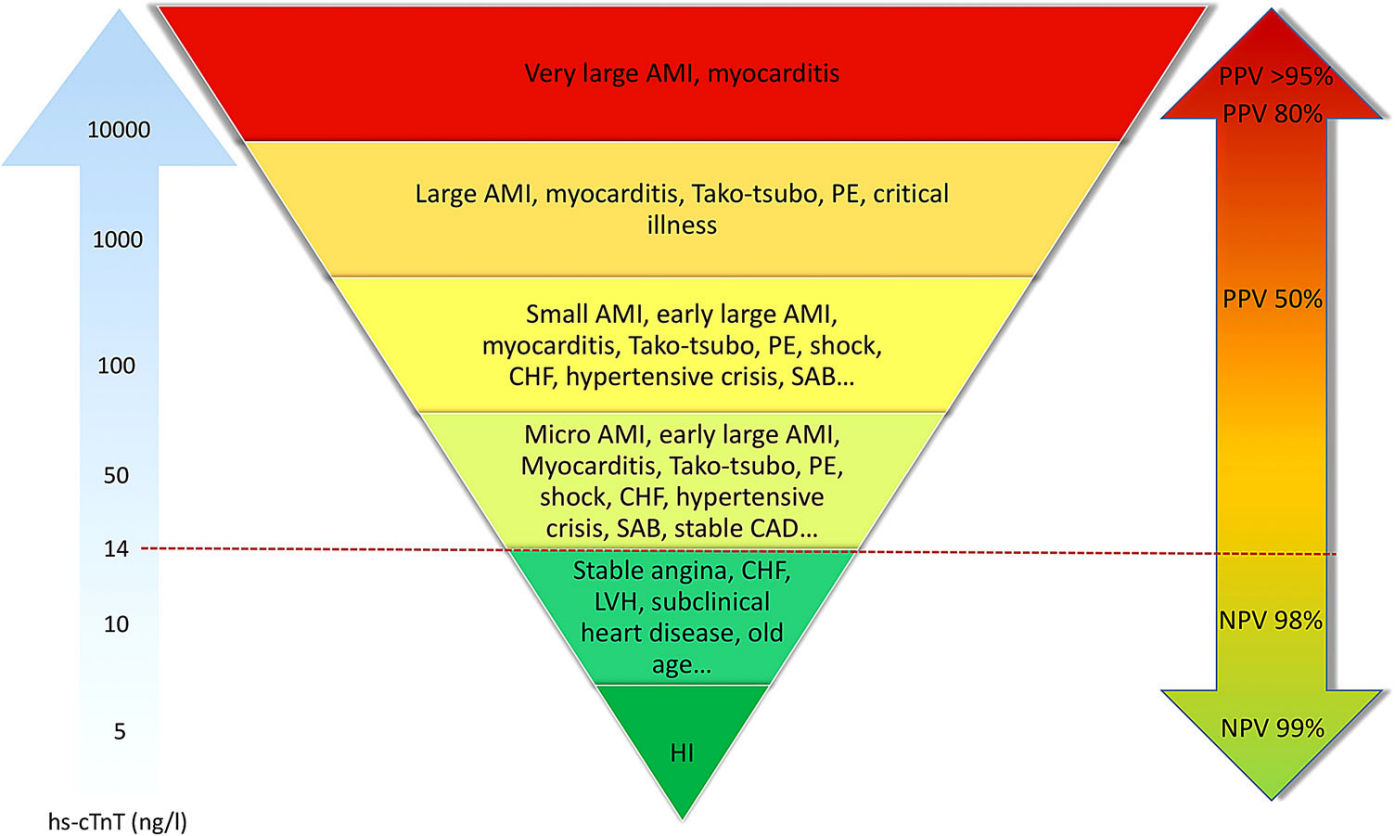
High-sensitivity cardiac troponin as a quantitative marker. *AMI* acute myocardial infarction, *CAD* coronary artery disease, *CHF* congestive heart failure, *HI* healthy individual, *LVH* left ventricular hypertrophy, *PE* pulmonary embolus, *SAB Staphylococcus aureus* bacteraemia. The lower the level of hs-cTn, the higher the negative predictive value (NPV) for the presence of AMI. The higher the level of hs-cTn, the higher the positive predictive value (PPV) for the presence of AMI. Levels just above the 99th percentile have a low PPV for AM

There is a risk of misinterpretation of elevated troponin results. Almost 13% of patients presenting with raised hs-cTn and chest pain eventually prove not to have ACS [34]. Hs-cTn can be detected in patients with various cardiac and non-coronary cardiovascular co-morbidities (Fig. 3).

## High-sensitivity cardiac troponin elevation in chronic kidney disease

More sensitive cTn assays also maintain high diagnostic accuracy in patients with renal dysfunction when assay-specific higher optimal cutoff levels are used [35]. Currently, there is no consensus on whether diagnostic criteria for AMI should differ for patients with and without impaired renal function [36]. The high prevalence of persistently elevated more sensitive cTn levels in patients with chronic kidney disease (CKD) cannot primarily be explained by reduced renal clearance alone [36]. The etiology of persistent troponin elevation in CKD remains incompletely explained and controversial. The underlying process appears to be multifactorial related to both increased subclinical cardiac damage (uremic toxicity, ischemic heart disease, heart failure or hypertensive heart disease) and decreased renal clearance in this population [37, 38]. The predictive value of cTn assays is maintained in patients with CKD [39]. Troponin elevation in patients with CKD should thus be taken seriously, and not merely be discounted as the result of decreased renal clearance.

## Use of high-sensitivity cardiac troponin in clinical practice

### Acute versus chronic elevation of troponin rise

Both the European Society of Cardiology (ESC) guidelines on the definition of AMI and suspected ACS endorse the use of hs-cTn assays [11, 40]. The obvious clinical advantage of hs-cTn assays is the shorter time interval to the second measurement of hs-cTn [24]. According to the current guidelines, a 3 h rule out protocol can now be used [11, 41].

To maintain a high specificity, it is important to distinguish acute from chronic hs-cTn elevation. Acute causes of hs-cTn elevation are associated with a corresponding significant rise or fall of hs-cTn. Acute cardiomyocyte injury causes a steep release of troponins, such as in AMI, shock, myocarditis, pulmonary embolus, Tako-tsubo (stress-induced) cardiomyopathy. Chronic, stable elevations of hs-cTn at or above the 99th percentile without a significant rise or fall are common in patients with structural heart disease [11]. In these cases, increased ventricular wall tension is thought to cause direct myofibrillar filament damage and an increase in programmed cell death, both of which contribute to hs-cTn release [42]. This has been observed in patients with left ventricular hypertrophy, valvular heart disease, stable congestive heart failure, pulmonary hypertension, stable angina or other forms of clinically stable cardiomyopathy. Table 2 outlines some common clinical causes of cTn elevation. Figure 3 depicts a quantitative approach to hs-cTnT elevation.

**Table 2:** Other causes of troponin elevation not secondary to acute myocardial infarction (AMI)

Oxygen demand mismatch (in the absence of AMI)
Tachy-/brady-arrhythmias
Hypertensive crisis
Anemia
Hypovolemia or hypotension
Aortic dissection or aortic valve disease
Hypertrophic cardiomyopathy
Strenuous exercise
Direct myocardial damage
Cardiac contusion
Cardiac procedures: cardioversion, pacing, ablation, endomyocardial biopsy
Cardiac infiltrative disorders, e.g., amyloidosis, haemochromatosis, sarcoidosis, sclerodermia
Chemotherapy, e.g., adriamycin, 5-fluorouracil, trastuzumab
Myocarditis or pericarditis
Cardiac transplantation (immune-mediated reactions)
Myocardial strain
Severe congestive heart failure: acute and chronic
Pulmonary embolism
Pulmonary hypertension or COPD
Accumulation of troponin in plasma
Acute/chronic renal dysfunction
Systemic processes
Sepsis
Systemic inflammatory processes
Burns, if affecting >30% of body surface area
Hypothyroidism
Snake venoms
Neurological disorders
Intracerebral hemorrhage or stroke
Seizures

### High-sensitivity cardiac troponin kinetics with serial testing

To differentiate acute from chronic troponin elevation and to maintain a high specificity, clinical evaluation (pre-test probability) and serial testing of hs-cTn are warranted. Various rule-in and rule-out algorithms have been proposed using different time points and cutoff values, including the question whether absolute or relative hs-cTn changes should be used. The use of any of these change criteria increases specificity (at the price of slightly decreased sensitivity) [43]. Despite the excellent performance of hs-cTn assays in the distinction of patients with AMI from patients with non-coronary artery cardiac diseases (such as hypertensive urgency/emergency, acute heart failure, and cardiac dysrhythmia), evidence for serial testing to improve specificity for type 1 myocardial infarction (ischemia from a primary coronary event) versus type 2 myocardial infarction (secondary to ischemia from a supply-and-demand mismatch) is limited. Most studies that have evaluated whether specificity can be increased by serial troponin testing have included type 1 and type 2 MI combined [44]. So far, the only study to evaluate the utility of serial testing to distinguish the more common type 2 from type 1 demonstrates no added advantage of serial testing of conventional troponin I [45]. In summary, while serial hs-cTn excellently distinguishes between acute and chronic myocardial injury, it remains uncertain whether it also helps in the distinction between type 1 and type 2 myocardial infarction.

Optimal cutoffs for (absolute and relative) changes and the earliest time points of the second hs-cTn measurement will have to be determined for each assay and clinical background (pre-test probability, rule-in vs. rule-out of AMI, special patient populations such as the elderly, patients with severe renal dysfunction, diabetic patients) separately and are the subject of current research.

### Rule-in and rule-out algorithms for AMI

**Fig. 4:**
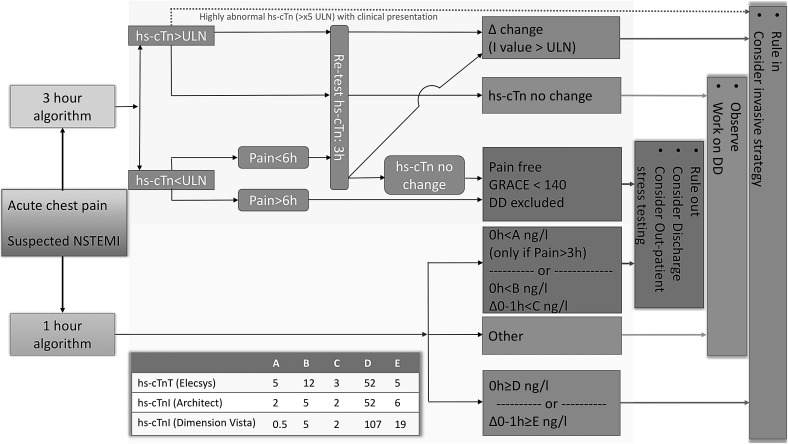
Algorithm for rapid early rule-in and rule-out of acute myocardial infarction with high-sensitivity cardiac troponin assays, adapted from [40]. It is generally recommended to use the 3-h algorithm. In cases of high pre-test probability for NSTEMI and if chest pain onset >3 h, a 1-h algorithm has now been proposed with assay-specific hs-cTn cutoff levels. Any algorithm should always be used in conjunction with clinical assessment and 12-lead ECG. Repeat blood sampling may be deemed necessary in cases of ongoing or recurrent chest pain. *GRACE* “Global Registry of Acute Coronary Events score”, *hs-cTn* high-sensitivity cardiac troponin, *ULN* upper limit of normal, 99th percentile of healthy controls, *D* change is dependent on assay, *DD* differential diagnosis

Figure 4 illustrates two algorithms (3- and 1-h) for rapid early rule-in and rule-out of acute myocardial infarction with hs-cTn assays based on current guidelines [40]. The latest guidelines recommend using the 3-h algorithm (Fig. 4). In cases of high pre-test probability for NSTEMI and if chest pain onset >3 h, a 1-h algorithm has been recommended when hs-cTn assays with a ‘validated algorithm’ are available (Elecsys, Architect, Dimension Vista). Assay-specific cutoff values are now available making use of the continuous, quantitative information of hs-cTn assays and the concept that the probability of myocardial infarction increases with increasing hs-cTn values. Additional blood sampling after 3 h in patients with strong clinical suspicion of AMI but no significant rise or fall of hs-cTn may nevertheless still be warranted: patients with AMI whose hs-cTn is serially measured around its peak may, e.g., not show any “significant” change. Any hs-cTn algorithm should always be used in conjunction with clinical assessment of pre-test likelihood of coronary artery disease, chest pain history and a 12-lead ECG.

### Gender-specific troponin thresholds

Due to a more atypical presentation, women remain a challenging group with regard to the diagnosis of myocardial infarction. Studies regarding gender-specific lower thresholds for women for the diagnosis of acute myocardial infarction have not shown consistent results: Shah et al. proposed that women-specific lower diagnostic thresholds for hs-cTn may double the diagnosis of myocardial infarction in women, and identify those at high risk of re-infarction and death [46]. On the other hand, in a larger recent study by Giménez et al., gender-specific troponin thresholds have not improved diagnostic accuracy, and it has been proposed that the 99th percentile should remain the standard of care for both genders [47].

### Outlook

Accelerated diagnostic protocols using hs-cTn assays have now been widely proposed [48], endorsed by current guidelines [40], and are being adopted in clinical practice in many countries with the exception of the United States where hc-cTn assays are still not available. Whereas ESC guidelines currently propose rapid algorithms for AMI (0 h/3 h or 0 h/1 h) using hs-cTn assays based on large validation cohorts, the AHA/ACC guidelines still recommend using conventional troponin assays and the longer 6 h troponin serial measurement [49].

More recently, several studies have tested lower cutoffs for hs-cTn for ruling out AMI [46, 48, 50]. Lower thresholds of hs-cTn I have better sensitivity than current standard thresholds [50]. The use of lower thresholds than the 99th percentile and very low thresholds below the limit of detection [50] has a very high NPV for AMI, and might be helpful in the early discharge of patients.

## Conclusion

Cardiac biomarkers for diagnosis of AMI have become more and more sensitive in recent decades. The currently used hs-cTn assays are highly valuable for rule-in and rule-out of AMI. International guidelines have been published for appropriate use of hs-cTn. Acute changes in hs-cTn complement the quantitative information provided by hs-cTn, and help in the differential diagnosis of diseases with chronic, stable troponin elevations vs. diseases with acute troponin elevations and acute cardiac damage. Serial testing of hs-cTn does not differentiate Type 1 from Type 2 myocardial infarction and, hence, integrating the results of hs-cTn measurements with robust clinical assessment remains the optimal approach.

## References

[1] Thygesen K, Alpert JS, Jaffe AS (2012). Third universal definition of myocardial infarction. Eur Heart J.

[2] Kavsak PA, MacRae AR, Lustig V (2006). The impact of the ESC/ACC redefinition of myocardial infarction and new sensitive troponin assays on the frequency of acute myocardial infarction. Am Heart J.

[3] Gamble JHP, Carlton E, Orr W, Greaves K (2013). High-sensitivity troponin: six lessons and a reading. Br J Cardiol.

[4] White HD (2011). Pathobiology of troponin elevations: do elevations occur with myocardial ischemia as well as necrosis?. J Am Coll Cardiol.

[5] Agewall S, Giannitsis E, Jernberg T, Katus H (2011). Troponin elevation in coronary vs non-coronary disease. Eur Heart J.

[6] LaDue JS, Wroblewski F, Karmen A (1954). Serum glutamic oxaloacetic transaminase activity in human acute transmural myocardial infarction. Science.

[7] World Health Organization Expert Committee (1959) Hypertension and coronary heart disease: classification and criteria for epidemiological studies. First report of the expert committee on cardiovascular diseases and hypertension. WHO Tech Rep Ser 168 13648449

[8] Panteghini M (1995). Enzyme and muscle diseases. Curr Opin Rheumatol.

[9] Danese E, Montagnana M (2016). An historical approach to the diagnostic biomarkers of acute coronary syndrome. Ann Transl Med.

[10] Gibler WB, Gibler CD, Weinshenker E (1987). Myoglobin as an early indicator of acute myocardial infarction. Ann Emerg Med.

[11] Thygesen K, Mair J, Giannitsis E (2012). How to use high-sensitivity cardiac troponins in acute cardiac care. Eur Heart J.

[12] Eggers KM, Oldgren J, Nordenskjöld A, Lindahl B (2004). Diagnostic value of serial measurement of cardiac markers in patients with chest pain: limited value of adding myoglobin to troponin I for exclusion of myocardial infarction. Am Heart J.

[13] Dolci A, Panteghini M (2006). The exciting story of cardiac biomarkers: from retrospective detection to gold diagnostic standard for acute myocardial infarction and more. Clin Chim Acta.

[14] World Health Organization (1979) Report of the Joint International Society and Federation of Cardiology/World Health Organization Task Force on Standardization of Clinical Nomenclature. Nomenclature and criteria for diagnosis of ischemic heart disease. Circulation 59:607–609 10.1161/01.cir.59.3.607 761341

[15] Ebashi S, Kodama A (1965). A new protein factor promoting aggregation of tropomyosin. J Biochem.

[16] Katus HA, Remppis A, Looser S (1989). Enzyme linked immuno assay of cardiac troponin T for the detection of acute myocardial infarction in patients. J Mol Cell Cardiol.

[17] Jaffe AS, Landt Y, Parvin CA (1996). Comparative sensitivity of cardiac troponin I and lactate dehydrogenase isoenzymes for diagnosing acute myocardial infarction. Clin Chem.

[18] Balk EM, Ioannidis JP, Salem D (2001). Accuracy of biomarkers to diagnose acute cardiac ischemia in the emergency department: a meta-analysis. Ann Emerg Med.

[19] Ooi DS, Isotalo PA, Veinot JP (2000). Correlation of antemortem serum creatine kinase, creatine kinase-MB, troponin I, and troponin T with cardiac pathology. Clin Chem.

[20] Takeda S, Yamashita A, Maeda K, Maéda Y (2003). Structure of the core domain of human cardiac troponin in the Ca(2+)-saturated form. Nature.

[21] Freda BJ, Tang WHW, Van Lente F (2002). Cardiac troponins in renal insufficiency: review and clinical implications. J Am Coll Cardiol.

[22] Reichlin T, Hochholzer W, Bassetti S (2009). Early diagnosis of myocardial infarction with sensitive cardiac troponin assays. N Engl J Med.

[23] Apple FS, Ler R, Murakami MM (2012). Determination of 19 cardiac troponin I and T assay 99th percentile values from a common presumably healthy population. Clin Chem.

[24] Mueller C (2014). Biomarkers and acute coronary syndromes: an update. Eur Heart J.

[25] Carlton EW, Cullen L, Than M (2015). A novel diagnostic protocol to identify patients suitable for discharge after a single high-sensitivity troponin. Heart.

[26] Weber M, Bazzino O, Navarro Estrada JL (2011). Improved diagnostic and prognostic performance of a new high-sensitive troponin T assay in patients with acute coronary syndrome. Am Heart J.

[27] Shah ASV, Anand A, Sandoval Y (2015). High-sensitivity cardiac troponin I at presentation in patients with suspected acute coronary syndrome: a cohort study. Lancet.

[28] Zhelev Z, Hyde C, Youngman E (2015). Diagnostic accuracy of single baseline measurement of Elecsys Troponin T high-sensitive assay for diagnosis of acute myocardial infarction in emergency department: systematic review and meta-analysis. BMJ.

[29] Shah ASV, Newby DE, Mills NL (2013). High sensitivity cardiac troponin in patients with chest pain. BMJ.

[30] Røysland R, Kravdal G, Høiseth AD (2012). Cardiac troponin T levels and exercise stress testing in patients with suspected coronary artery disease: the Akershus Cardiac Examination (ACE) 1 study. Clin Sci (Lond).

[31] Sabatine MS, Morrow DA, de Lemos JA (2009). Detection of acute changes in circulating troponin in the setting of transient stress test-induced myocardial ischaemia using an ultrasensitive assay: results from TIMI 35. Eur Heart J.

[32] Carlson RJ, Navone A, McConnell JP (2002). Effect of myocardial ischemia on cardiac troponin I and T. Am J Cardiol.

[33] Jeremias A, Gibson CM (2005). Narrative review: alternative causes for elevated cardiac troponin levels when acute coronary syndromes are excluded. Ann Intern Med.

[34] Mueller M, Vafaie M, Biener M (2013). Cardiac troponin T: from diagnosis of myocardial infarction to cardiovascular risk prediction. Circ J.

[35] Twerenbold R, Wildi K, Jaeger C (2015). Optimal cutoff levels of more sensitive cardiac troponin assays for the early diagnosis of myocardial infarction in patients with renal dysfunction. Circulation.

[36] Newby LK, Jesse RL, Babb JD (2012). ACCF 2012 expert consensus document on practical clinical considerations in the interpretation of troponin elevations: a report of the American College of Cardiology Foundation task force on Clinical Expert Consensus Documents. J Am Coll Cardiol.

[37] Parikh RH, Seliger SL, deFilippi CR (2015). Use and interpretation of high sensitivity cardiac troponins in patients with chronic kidney disease with and without acute myocardial infarction. Clin Biochem.

[38] Dikow R, Hardt SE (2012). The uremic myocardium and ischemic tolerance: a world of difference. Circulation.

[39] Haaf P, Reichlin T, Twerenbold R (2014). Risk stratification in patients with acute chest pain using three high-sensitivity cardiac troponin assays. Eur Heart J.

[40] Roffi M, Patrono C, Collet J-P (2015). 2015 ESC Guidelines for the management of acute coronary syndromes in patients presenting without persistent ST-segment elevation. Eur Heart J.

[41] Hamm CW, Bassand J-P, Agewall S (2011). ESC Guidelines for the management of acute coronary syndromes in patients presenting without persistent ST-segment elevation: the Task Force for the management of acute coronary syndromes (ACS) in patients presenting without persistent ST-segment elevatio. Eur Heart J.

[42] Logeart D, Beyne P, Cusson C (2001). Evidence of cardiac myolysis in severe nonischemic heart failure and the potential role of increased wall strain. Am Heart J.

[43] Reichlin T, Irfan A, Twerenbold R (2011). Utility of absolute and relative changes in cardiac troponin concentrations in the early diagnosis of acute myocardial infarction. Circulation.

[44] Haaf P, Drexler B, Reichlin T (2012). High-sensitivity cardiac troponin in the distinction of acute myocardial infarction from acute cardiac noncoronary artery disease. Circulation.

[45] Sandoval Y, Thordsen SE, Smith SW (2014). Cardiac troponin changes to distinguish type 1 and type 2 myocardial infarction and 180-day mortality risk. Eur Heart J Acute Cardiovasc Care.

[46] Shah ASV, Griffiths M, Lee KK (2015). High sensitivity cardiac troponin and the under-diagnosis of myocardial infarction in women: prospective cohort study. BMJ.

[47] Rubini Giménez M, Twerenbold R, Boeddinghaus J (2016). Clinical effect of sex-specific cutoff values of high-sensitivity cardiac troponin T in suspected myocardial infarction. JAMA Cardiol.

[48] Neumann JT, Sörensen NA, Schwemer T (2016). Diagnosis of myocardial infarction using a high-sensitivity troponin I 1-hour algorithm. JAMA Cardiol.

[49] Rodriguez F, Mahaffey KW (2016). Management of patients with NSTE-ACS. J Am Coll Cardiol.

[50] Carlton E, Greenslade J, Cullen L (2016). Evaluation of high-sensitivity cardiac troponin I levels in patients with suspected acute coronary syndrome. JAMA Cardiol.

